# To what degree does the missing-data technique influence the estimated growth in learning strategies over time? A tutorial example of sensitivity analysis for longitudinal data

**DOI:** 10.1371/journal.pone.0182615

**Published:** 2017-09-13

**Authors:** Liesje Coertjens, Vincent Donche, Sven De Maeyer, Gert Vanthournout, Peter Van Petegem

**Affiliations:** 1 Psychological Sciences Research Institute, Université Catholique de Louvain, Louvain-la-Neuve, Belgium; 2 Department of Educational Sciences, Faculty of Social Sciences, University of Antwerp, Antwerp, Belgium; 3 Artesis Plantin University College, Antwerp, Belgium; Tilburg University, NETHERLANDS

## Abstract

Longitudinal data is almost always burdened with missing data. However, in educational and psychological research, there is a large discrepancy between methodological suggestions and research practice. The former suggests applying sensitivity analysis in order to the robustness of the results in terms of varying assumptions regarding the mechanism generating the missing data. However, in research practice, participants with missing data are usually discarded by relying on listwise deletion. To help bridge the gap between methodological recommendations and applied research in the educational and psychological domain, this study provides a tutorial example of sensitivity analysis for latent growth analysis. The example data concern students’ changes in learning strategies during higher education. One cohort of students in a Belgian university college was asked to complete the Inventory of Learning Styles–Short Version, in three measurement waves. A substantial number of students did not participate on each occasion. Change over time in student learning strategies was assessed using eight missing data techniques, which assume different mechanisms for missingness. The results indicated that, for some learning strategy subscales, growth estimates differed between the models. Guidelines in terms of reporting the results from sensitivity analysis are synthesised and applied to the results from the tutorial example.

## Introduction

In the educational research domain, longitudinal design is relied upon to assess, for example, how achievement goals evolve during the transition from elementary to secondary school, how reading comprehension evolves after an intervention, or how student learning changes during higher education [1]. The data gathered in such longitudinal designs almost invariably contain a certain amount of missing data [2–4].

This missing data is a major issue in educational and psychological research. Peugh and Enders [5] found that, in longitudinal studies in these research domains, on average 9.78% of the data was missing, and this could grow to a maximum of 67%. In the study by Rombach and colleagues [6], the percentage of missing data ranged from 1% to over 70%, with a median percentage of 25%.

In determining the impact of these missing data on the results obtained, Rubin [7] considered the mechanism generating the missing data (i.e., the reason why the data are missing, [8]) to be crucial. Three mechanisms generating missing data are identified; missing completely at random (MCAR), missing at random (MAR) and missing not at random (MNAR) [7, 9]. Depending upon the mechanism generating the missing data, the literature describes different techniques to deal with these eventualities [10, 11].

However, for practitioners using non-simulated data, the mechanism generating missing data is most likely hidden: it cannot be discerned if data are MAR or MNAR. In the light of this, it is advocated that researchers conduct a sensitivity analysis to gauge the stability of the models’ results to missing data techniques, assuming MAR or MNAR [2, 12, 13].

Up to the present, there is an extensive body of simulation research on sensitivity analysis with missing data [14, 15–18]. Next to this, a number of studies have (additionally) used non-simulated data to exemplify sensitivity analysis with missingness being possibly MNAR, predominantly in the domain of (bio)medicine [19, 20–22], but also in the psychological and educational domain [23, 24]. However, sensitivity analysis is seldom applied to longitudinal studies in the psychological and educational domains ([12, 13, 16]; for an exception see [25]).

In this respect, the limited number of studies on sensitivity analysis aimed at a non-statistical audience that take a tutorial focus, is not helpful. Though very clearly written tutorials on missing data analysis in the psychological and educational domain can be found [26–29], the techniques used in these studies are limited to techniques assuming MCAR and MAR. The present study aims to provide a tutorial example with regard to conducting sensitivity analysis using various missing data techniques, amongst which are also techniques assuming MNAR.

The results on analyses using different missing data techniques may contradict one another [2, 21, 30, 31]. When this is the case, how to report the results may appear unclear to a non-statistical researcher. Thus, the second aim of the study is to provide a summary of the guidelines for practice in reporting on the results from sensitivity analysis, and to apply these guidelines to the tutorial example given.

The remainder of the article is organized as follows; the first section of the introduction will detail the mechanisms generating missingness and sections 2 to 4 will provide the background on different techniques, assuming MCAR, MAR and MNAR respectively. We aim for a conceptual overview of the mechanisms and different techniques applied to analysing change over time with longitudinal data for a non-statistical audience. For more detail on these techniques and their mathematical underpinnings with regard to missing data, we would refer a novice practitioner initially to Enders [30] and Graham [32]. Even more detail can be found in the references cited below. The method section provides more detail on the data used for the tutorial example, and on how the different statistical techniques were applied. Then the results from the sensitivity analysis are discussed. Finally, in the discussion section, we provide guidelines for the practice of reporting results from a sensitivity analysis, and we apply these guidelines to the tutorial example.

### Mechanisms generating missingness

The literature discerns three mechanisms by which missing data could occur [7, 9, 11]. Though they are applicable to all data collections involving missing data, we illustrate them here in the case of longitudinal data. Given that the mechanism abbreviations are easily mistaken for one another, Table 1 provides a short summary.

**Table 1:** Detail on abbreviations regarding missingness.

Abbreviation	Full term	Probability of missingness is related to	Technique
MCAR	Missing Completely at Random	chance	1. Listwise deletion (LD)
MAR	Missing at Random	variable(s) in the study (e.g., score at previous wave, demographic characteristic)	2. Maximum Likelihood (ML) 3. Multiple Imputation (MI) 4. ML with auxiliary variables (MLaux) 5. MI with auxiliary variables (MIaux)
MNAR	Missing Not at Random	the unobserved change over time (e.g., change in scores between waves)	Pattern mixture (PM) models: 6. Hedeker & Gibbons (H&G); 7.-8. Models with identifying restrictions

Firstly, missingness may be MCAR. This mechanism hold when “…the cases for which the data are missing can be thought of as a random sample of all the cases” [32] (p. 552). An example from the learning strategies domain could be that a student misses a data collection due to influenza. Yet, as several authors have pointed out, to assume longitudinal data are *solely* MCAR is stringent, and unlikely to hold up in practice [8, 33, 34].

A second mechanism generating missing data holds when the probability of missingness is related to one or several of the variables collected in the study, such as the score with regard to a previous wave. When this relationship is controlled, there is no further relationship between the missing values and the pattern of missingness. This mechanism is labelled MAR [7, 9, 35]. An example would be when students who were scoring higher on surface processing at the first wave were found to have a higher chance of dropping out of higher education, and therefore are found to be absent at the second wave.

Thirdly, the chance of being missing can depend upon the—unobserved—missing value (outcome-related missing data or MNAR, [9, 11]). This would be the case if students who decreased their deep processing from wave one to wave two, were more prone to dropping out of higher education prior to the second wave of data collection. The unobserved change over time in deep processing then predicts the chance of being missing.

Please note that the difference between the MAR and MNAR mechanisms depends on whether or not the missing data is related to unobserved values in deep processing, after controlling for other variables in the dataset [11, 32]. With MAR, “…there is no relationship between the propensity for missing data on Y and the values of Y after partialling out other values” [30] (p. 6), such as observed scores on deep processing at previous waves. With MNAR, on the other hand, the change over time in deep processing leading to the missing data is unobserved, even after controlling for the observed values on deep processing.

In non-simulated longitudinal data, all three mechanisms that generate missing data may be present [30]. Some missing data can be due to reasons in line with MCAR, whilst other missing data are caused by MAR or MNAR mechanisms. Whether or not missing data are MCAR can be tested using the independent samples *t*–test or Little’s MCAR test [10, 30, 36]. When these tests provide significant results, the MCAR assumption is rejected. Consequently, missing data is also MAR. Note that the absence of significant results does not support the MCAR assumption. First, missing data may still be due to MAR but, perhaps due to low statistical power (e.g., small N), no significant results were detected. Second, missingness caused by an MNAR mechanism may also lead to non-significant results [30].

In contrast to the MCAR assumption, neither the MAR nor the MNAR assumption can be tested, due to the fact that the answer lies within the absent data [11, 12]. For this reason, it is recommended that the researcher gauges the sensitivity of the parameter estimates to the mechanism for the missing data [2, 13, 14, 16]. The results from techniques assuming MAR and MNAR should then be compared. If "…different methods result in different parameter estimates of the longitudinal model, this may be an indication that the missing data mechanism is an important element in describing the data" [37] (p. 254).

Prior to discussing missing data techniques assuming MAR or MNAR, we will first discuss a tradiional method for handling missing data, namely listwise deletion (LD). Up to the present, traditional methods and more specifically, listwise deletion, are frequently used in educational research and in psychological research [e.g., 44% of longitudinal studies relied on techniques assuming MCAR, [18], 33% of studies relied on listwise deletion, [6]).

### Technique assuming an MCAR mechanism: Listwise deletion

Listwise deletion (LD) implies that cases with missing data are discarded from the analysis. When data are MCAR and the sample is sufficiently large, this technique has been shown to produce adequate parameter estimates. However, when the MCAR assumption is not met, listwise deletion will result in bias [28, 38]. Please note that for the remainder of the paper and in line with Enders [30] and Schafer and Graham [38], we will abbreviate this reasoning to ‘listwise deletion assumes an MCAR mechanism’.

An example may help clarify why, when the MCAR assumption is not met, listwise deletion will result in bias. Suppose that the chance of having missing data is related to students’ deep learning: students who do respond at the different waves tend to score more highly in terms of deep learning. Analysis on the group of students for whom full data is available will omit proportionally more students with lower deep learning scores. Hence, the average score for deep learning at the first wave (i.e., the mean intercept) will be overestimated. Moreover, the variance in the intercept of deep learning will be underestimated. As a consequence, as succinctly stated by Wothke [39], listwise deletion yields “…very precise estimates of exactly the wrong parameter” (p. 230).

Even if the MCAR assumption is not disproven, LD is judged to be suboptimal, due to the lower power caused by the reduction in sample size [5, 10]. The MCAR technique does not use the available data efficiently [39, 40]. Therefore, methodologists and the APA Task Force on Statistical Inference strongly advised against the use of LD [9, 30], judging it to be “…among the worst methods for practical applications” [41] (p. 598).

### Techniques assuming an MAR mechanism: Maximum likelihood, multiple imputation and the inclusion of auxiliary variables

We will discuss two frequently used techniques assuming MAR (see Table 1). The first one, Maximum Likelihood (ML), estimates the parameters which are most likely to have produced the sample data by trying out different values for these parameters (For a detailed description, see [30]). With missing data, this is not different, just more complex. For each respondent, the computation of the log-likelihood is based on the available data. If a respondent provided a set of complete data, the calculation of the log-likelihood is based on all data. For a respondent with missing data on one variable, the log-likelihood is calculated for all parameters for which the respondent does have data. Subsequently, the log-likelihood for all respondents is added together. In order to find the most likely parameter estimates, these calculations are made for different estimates of the population parameters. By including the respondents with missing data, the parameter estimates for which they provide data is fine-tuned, leading to more correct parameter estimates for the variables on which they did not provide data [10, 11, 30, 38].

A second technique, assuming MAR, is multiple imputation (MI), which involves three phases [38, 42, 43]. In the imputation phase, *m* (e.g., 100) complete datasets are generated by filling in the missing values with different plausible estimates. These estimates are derived from regression equations based upon the complete data, to which a normally distributed residual term was added, to take into account the uncertainty concerning the estimate. In the analysis phase, analysis is done on each of the *m* complete datasets. In the third phase, the parameter estimates and the standard errors are pooled. Parameter estimates are estimated as the mean over the *m* datasets, whilst their standard errors are computed by taking into account both the variance within datasets (within variance) and between datasets (between variance, [10, 42]).

In both the ML and MI techniques, auxiliary variables can be included (MLaux and MIaux). Auxiliary variables are included in order to estimate (ML) or fill in (MI) missing values in a more informed or accurate way. Consequently, by predicting some of the missingness, an MNAR missingness situation could be turned into an MAR situation [10, 31, 44]. For this reason, if auxiliary variables are available, the MLaux and MIaux techniques are preferred over ML and MI [35, 43].

Two types of variables are of interest as auxiliary variables [35, 43]. Firstly, variables that predict missingness can be informative. If, for example, girls are more likely to participate, gender can be used. Secondly, variables correlating moderately-to-strongly with the variables under consideration are of interest. If deep approach and grade point averages are correlated, the latter can be included when estimating or imputing the missing data for the deep approach variable.

### Techniques assuming an MNAR mechanism: Pattern mixture models and selection models

There are two families of MNAR models: pattern mixture (PM) models and selection models [11, 31, 45–47]. The former divide the sample into subgroups depending on missing data patterns (e.g., a group with complete information, a group with information only at the first wave). Next, the parameter estimates are estimated for each of the subgroups, allowing for the examination of how the results vary by group. Finally, the results of the different models are put together [31, 37]. A second family consists of selection models which estimate the probability of missingness and the parameters simultaneously in one model [31, 48].

Both families of models rely upon a number of untestable assumptions. For the selection models, small departures from the multivariate normality assumption can have a serious bias effect on the results [21, 45, 47]. For the PM models, restrictions have to be imposed in order to allow the models to be estimated for all subgroups [11]. Yet, these last assumptions are explicit [47], and different restrictions can be used to assess the sensitivity of the results to them [12, 23, 24]. Moreover, tutorial examples of selection models in the psychological educational domain are available in the literature [8, 24]. Given that few practical examples are available in educational and psychological research on sensitivity analysis using PM models, we have focused our study on these models assuming MNAR. More specifically, we selected four PM models.

The first PM model was the Hedeker and Gibbons (H&G) model [49], which assesses the parameter estimates for respondents with complete data (subgroup 1) and for respondents with incomplete data (subgroup 2). As Little [47] noted, “…pattern-mixture models are chronically underidentified” (p. 125), meaning that for certain groups the model cannot be estimated without borrowing information from other subgroups. For example, if the respondents of subgroup 2 were to have only information on the first two of three waves, the change over time can be estimated but, due to the missing third wave, there is a lack of data to estimate the variance and covariance of this subgroup [30]. Therefore, in the example given, the H&G model assumes that the variance and covariance of subgroup 1 can be relied upon to estimate the model for subgroup 2. After estimating the growth for both subgroups, the population parameters of interest are estimated by taking the proportion of each subgroup into account. To do so, the weighted average of the mean intercept and mean slope is estimated [49].

The other three PM models rely on more subgroups. For example, in a three wave study, three subgroups can be discerned: students with complete data (1), those who go missing after the second wave (2), and students who go missing after the first wave (3). Given that the students in subgroup 3 have only the initial data point, the mean value at the first wave can be estimated, but their change over time cannot. Once again, information has to be shared across subgroups to allow the change over time for subgroup 3 to be estimated. To model this, three types of identifying restrictions can be put into place (hence, three PM models, [19, 21, 50]).

In the *complete case* restriction [47], information from the group providing complete data (subgroup 1) is used: the change over time for subgroup 3 is equated to the estimates of the change over time for the complete cases (subgroup 1). Once the parameter estimates have been estimated for each of the three subgroups, the population parameters are derived by using the weighted average of the three groups. The *neighbouring case* restriction [21, 50] differs only in the fact that the change over time for subgroup 3 is equated to that of subgroup 2. The *available case* option [21, 50] consists of using the weighted average of the changes over time of subgroups 1 and 2.

Comparing the H&G model to the other three PM models, an advantage of the former is that longitudinal data can usually be divided into the two subgroups without the number of respondents per subgroup becoming too small [49]. A drawback of the H&G model is that all respondents with incomplete data are treated alike [12]. This may not always make sense intuitively. For example, students dropping out after the first year may be a different type of student than those dropping out after the second year. The other three PM models use more subgroups. Their drawback is deciding upon the most appropriate subgroups, and the need to prevent subgroups from becoming too small [12].

### This study

This study aims to provide a tutorial example of sensitivity analysis for latent growth analysis. To conduct such an analysis, the results from techniques assuming MAR (ML, MI, MLaux and MIaux) are checked against those assuming MNAR (PM models: H&G model, complete, neighbouring and available case restriction models). Given that LD is still frequently used in educational and psychological research [18, 26], we opted to include this technique also, which assumes an MCAR mechanism. In addition, a summary of the guidelines for practice in reporting on the results from sensitivity analysis are provided and applied to the tutorial example.

As example data, a non-simulated dataset on the change in students’ learning strategies during higher education was selected. Thus, similar to the situations of researchers confronted with missing data, the mechanism causing the missing data (MCAR, MAR or MNAR) and true parameter values are unknown. Yet, given that previous research findings indicate that study success and dropout in higher education is linked to students’ learning strategies [51, 52, 53], the MCAR assumption is unlikely to hold true. Moreover, in this data collection, there was a large time interval between the different waves. As such, change over time leading to dropout (and thus missing data) may have been unobserved, making the MNAR assumption plausible. This non-simulated dataset thus allows us to provide a genuine example of the use of sensitivity analysis when modelling growth in educational and psychological research.

## Method

### Ethics statement

For research in higher education, ethics approval and written consent is not required by Belgian law. The Law on Experiments on Humans (7th May 2004) obliges researchers to obtain ethical approval and consent for an experiment, whereby ‘experiment’ is defined as ‘‘…each study or research in which human persons are involved with the goal of developing appropriate knowledge for the performance of health professions” (‘‘elke op de menselijke persoon uitgevoerde proef, studie of onderzoek, met het oog op de ontwikkeling van de kennis eigen aan de uitoefening van de gezondheidszorgberoepen”, 2004050732/N, Article 2, paragraph 11). The current research is not related to the health professions and is therefore implicitly exempt from the need for ethical approval and written consent. We underline that participation at each wave was on a voluntary basis, and that the students, who were all adults, could stop their participation at any moment. There was no penalty for students who chose not to participate, nor were they rewarded for participation with, for example, student counselling regarding learning strategies. The confidentiality of the results was guaranteed by the research team.

### Participants

Data for one cohort of students in a Belgian university college is considered here. In March of the first academic year (from September to June), first-year students participated in the research. The same cohort was again questioned during May in both the second and third year of study. Students’ learning strategies consisted of cognitive processing and regulation activities, and are mapped using the Inventory of Learning Styles–Short Version (ILS-SV, [54]).

Three of the seven learning strategy subscales were selected from a tutorial perspective: the memorizing, lack of regulation and analysing subscales presented an array of possible outcomes from sensitivity analysis, which researchers may encounter in practice. By selecting a small number of subscales, the results and suggestions for practice can be presented in detail. Table 2 provides for the three subscales, the number of items, an example item and the reliability estimates.

**Table 2:** Three learning strategy subscales of the ILS-SV; number of items; item example (translated from Dutch); and reliability estimates.

Subscales	Items	Item example	α°
Memorizing	4	I learn definitions by heart and as literally as possible.	.68-.71
Lack of regulation	4	I confirm that I find it difficult to establish whether or not I have sufficiently mastered the course material.	.68-.73
Analysing	4	I study each course book chapter point by point and look into each piece separately.	.66-.70

° the lowest and highest α obtained for each of the three waves is given

### Latent growth analysis

**Fig 1:**
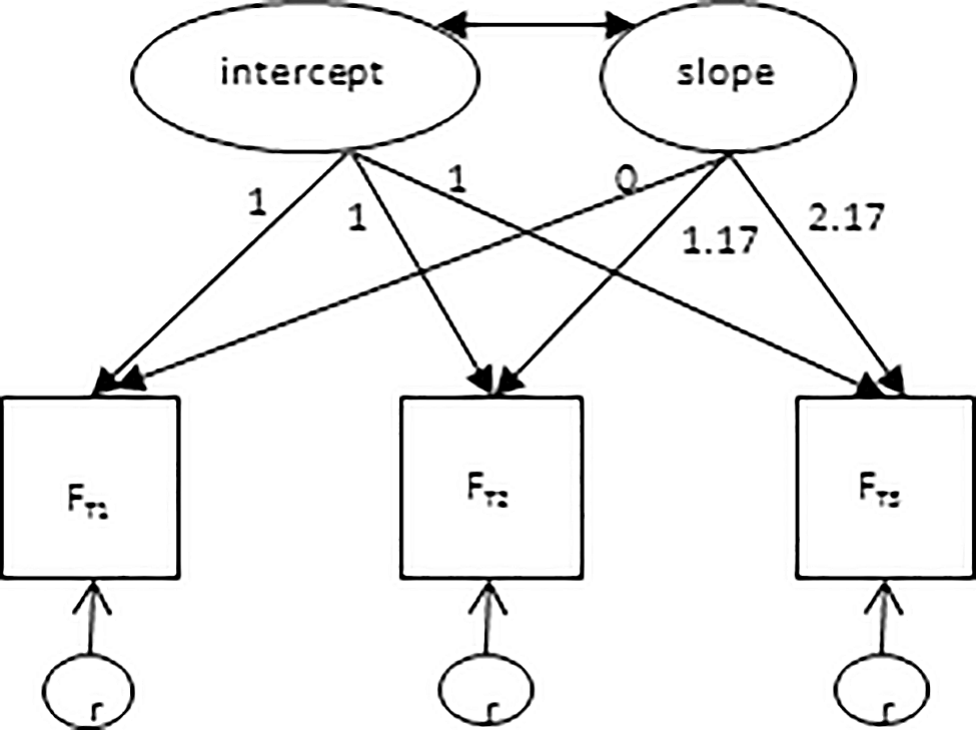
Latent growth model.

To gauge whether or not students increase their deep learning to the detriment of surface and unregulated learning, studies on learning strategies have relied upon latent growth analysis [55]. We use this technique here as well, given that it allows the estimation of MNAR models more easily than does multilevel analysis [30]. Fig 1 depicts such a model that, for each respondent in the dataset, estimates the growth in manifest subscale scores by an intercept and a slope [56, 57]. The mean intercept for all respondents then signifies the mean initial value for the subscale. The mean slope is estimated as the mean of the slopes of the respondents, and indicates the degree to which, on average, there is an increase or decrease in the subscale scores per unit of time (here, 12 months). Please note that due to data gathering at unequal time intervals (14 months between waves 1 and 2 and 12 months between waves 2 and 3), the values of the factor loadings for the slope have been adjusted to 0, 1.17 (being 14/12th) and 2.17, respectively [57, 58].

Three parameters are estimated next to the mean growth trajectory. Firstly, the intercept variance parameter expresses the degree to which students varied significantly in their initial level as measured by a learning strategy subscale. Secondly, the variance in the slope indicates the degree to which the students followed the general trend or deviated from one another. Thirdly, if both the intercept and slope variance proves significant, the covariance becomes a point of interest. This covariance indicates whether or not, on a learning strategy subscale, the students’ initial scores are related to their change over time [56, 57].

### Missing data and options to handle them

Table 3 provides detail on the missing data in the sample. There is a considerable amount of attrition. Of the cohort under consideration, 1,355 students were registered in the first year and 410 (30.3%) of those students continued in a non-delayed study trajectory into the third year. Due to unrestricted entrance into higher education in Belgium, it is commonplace that a large number of students stop during the first year or fail their exams (e.g., [59]). Next to attrition, there was wave non-response (i.e., respondents missing a wave but returning for a subsequent one). Given that students were questioned during lecture slots, the response rates were adequate: 76.1% of the eligible students participated in wave 1, descending to 66.6% in wave 3 (see Table 3). Note that some students participating in waves 2 and 3 had not participated in the first wave. Thirdly, there was a small number of item non-responses (i.e., respondents having participated in a data collection but having left one or multiple items unanswered). For example, at the first wave, six students did not complete all the items for the analysing subscale (see Table 3). Subscale scores were computed only if the student answered each of the four items for a learning strategy subscale. Therefore, item non-response was treated as wave non-response.

**Table 3:** Registration, participation and response rate per measurement wave.

	Wave 1	Wave 2	Wave 3
Number of registered students	1355	616	410
Number of respondents	1031	442	279
Response rate (%)	76.1	70.5	66.6
Number of respondents without item non-response_Memorizing and Lack of regulation_	1029	442	278
Number of respondents without item non-response_Analysing_	1025	440	275

In total, 21.8% (= 225/1029) of the students who participated in the first wave, provided complete information at each of the three waves (21.6% for the analysing subscale, 222/1025). This percentage was in line with (e.g., 21.5% [1]), or better than, other studies on the change in student learning strategies during higher education (7.5% and 6.5% in [60, 61] respectively).

We made use of all available data. The number of students providing data for at least one wave for the analysing subscale was 1,071, whilst 1,072 did so for the memorizing and lack of regulation subscale. The 283 students who did not provide data at any of the three waves were excluded from the analysis.

### Plan of analysis

Prior to analysing the growth over time for the memorizing, lack of regulation and analysing subscales, the MCAR assumption was tested using Little’s MCAR test in SPSS (see ‘Mechanisms generating missingness’). Subsequently, latent growth analysis was undertaken in M*plus* 6.1, using eight missing data techniques (see Table 1). Annotated syntax for each of these latent growth analyses are available in the S1 File and the datasets for the three scales can be found in S2–S4 Files.

Assuming MCAR, we estimated the latent growth model using LD (N_memorizing_ and N_lack of regulation_ = 225; N_analyzing_ = 222). We ran the other models on the sample of students providing data on at least one wave (N_analyzing_ = 1071 and N_memorizing_ and N_lack of regulation_ = 1072). We estimated two techniques assuming MAR, both without and with auxiliary variables: ML (through the EM algorithm, [22]); MI; MLaux; and MIaux. For the MI and MIaux models, we opted for 100 imputed datasets, given the large percentage of missing data. Moreover, a higher number of imputed dataset could increase the stability of the estimates and, since the latent growth model required only a short computational time, there was no drawback in including more datasets [62].

The following administrative data were available as auxiliary variables: gender; prior education (general, technical or vocational); study track in higher education; whether or not students had started the first year in that university college anew; whether or not they had followed a non-delayed study trajectory; and the grade point average for each year. Good auxiliary variables predict the chance of being missing or are correlated with the variables under consideration [28, 35, 43]. To examine the former, logistic regression was used to determine the variance explained by the auxiliary variables, as to whether or not students were missing at a wave. The results are given in Table 4. To examine the latter, regression was used to provide the explained variance in memorising, lack of regulation and analysing by the auxiliary variables (see Table 4). We note that none of the auxiliary variables was correlated at .40 or more with the scores at each of the three waves (as suggested by [35], while Table 4 denotes how many of the 15 auxiliary variables were correlated at higher than .10 with the scores at the three waves.

**Table 4:** Auxiliary variables.

	Explained variance in the chance of missingness by the auxiliary variables (Nagelkerke R^2^, in %)	Explained variance of the variables by the auxiliary variables (R^2^, in %)	Number of auxiliary variables correlated .10 or higher with the variable with missing data
Memorizing			
Wave 1	20	10.7	1
Wave 2	7.3	8.0	3
Wave 3	18.4	8.3	3
Lack of regulation			
Wave 1	20	14.4	5
Wave 2	7.3	17.3	5
Wave 3	18.4	20.4	7
Analysing			
Wave 1	20.1	10.5	2
Wave 2	7.4	9.6	2
Wave 3	18.4	12.5	3

There is debate in the literature on missing data with regard to which and how many auxiliary variables to include. The study by Collins et al. [35] suggested that there is no harm in including auxiliary variables that are little related to the variable under consideration. More recent simulation studies [63, 64] however, suggest that including too many auxiliary or specific kinds of auxiliary variables may, in fact, bias estimates and decrease precision. For this reason, Hardt et al. [64] recommended “…restricting the number of auxiliary variables to not more than 1/3 of the cases with complete data” (p. 10). In our case, with at least 222 cases with complete data, this would imply not including more than 74 variables. In addition, Hardt et al. [64] suggested that when categorical data are used [such as female or study track in our case), this ratio should be higher. Nonetheless, it is clear that with the 18 variables involved [3 scores and 15 auxiliary variables, 12 of which are categorical), we are well below this cut-off level. Therefore, all available data were included as auxiliary variables.

The techniques, assuming MNAR, consisted of the H&G model and three models with identifying restrictions. For the models with identifying restrictions, the main hurdle for a researcher is to decide how to form subgroups. In doing so, the research questions, previous research findings that may help understand the mechanism for missingness, as well as the size of the subgroups, are important [49]. If our aim is to understand how learning strategies change over time, and given the literature on the predictive value of these learning strategies on student dropout [53], we need to discern subgroups of attrition. For this, we relied upon administrative data regarding student enrolment in each of three academic years, to create true dropout patterns. Using ANOVA, we verified whether or not these three dropout patterns differed in terms of scores on the first two waves.

For the H&G model, students progressing normally throughout their three years of study (N = 395) were contrasted with their peers who dropped out (N = 677). For the identifying restriction models, we discerned three subgroups: students in a non-delayed trajectory (1, N = 395); those registered up to second year (2, N = 184) and those registered only in the first year (3, N = 493). Here, it is evident that the growth of subgroup 3 cannot be estimated. Therefore, three identifying restrictions can be used. In the complete case, the growth for subgroup 3 is equated to the growth of subgroup 1. In the neighbouring case, it was equated to the growth of subgroup 2. Lastly, with the available case restriction, we used the weighted average of subgroups 1 and 2.

Which of the three models with identifying restrictions is most likely to generate trustworthy results, should be argued for [30, 47]. Prior research findings can be relied upon to underpin the argumentation. In this particular dataset, based upon previous research in a similar context [53], we assume a relationship between learning strategies and dropout rates. Therefore, we are convinced once again in this particular dataset, that the assumption of the complete case model has less merit that those from the neighbouring case and from the available case models. As such, we opted not to estimate the complete case model.

## Results

First, the MCAR assumption was tested using Little’s MCAR test. The results indicated that the MCAR assumption could not be rejected for the memorizing subscale (Chi^2^ = 3.460, df = 9, p = .943), while it was rejected for the lack of regulation subscale (Chi^2^ = 62.183, df = 9, p < .001) and the analysing subscale (Chi^2^ = 32.226, df = 9, p < .001). Additionally, ANOVA were used to verify whether or not students in the three dropout groups, as discerned by administrative data, differed on the three learning strategy scales. The results indicate no significant differences between the dropout groups in terms of the memorizing scale (at wave 1: F(2,1026) = .889, p = .411, at wave 2: F(1,440) = .887, p = .347), whilst there are significant differences for the lack of regulation scale (at wave 1: F(2,1026) = 29.148, p < .001, at wave 2: F(1,440) = 8.252, p < .01). Students in group 1 (M = 2.61, SD = .86) scored significantly lower in terms of lack of regulation than their peers in groups 2 and 3 (respectively M = 2.89, SD = .78 and M = 3.04, SD = .80). At wave 2, students in group 1 scored significantly lower on the lack of regulation scale compared to students in group 2 (respectively, M = 2.52, SD = 0.86, M = 2.78, SD = .83). For the analysing scale, the results also indicated significant differences (at wave 1: F(2,1022) = 11.916, p < .001, at wave 2: F(1,438) = 4.320, p < .05). At the first wave, students in group 1 (M = 3.04, SD = .81) scored significantly higher compared to those in groups 2 and 3 (respectively M = 2.84, SD = .77 and M = 2.78, SD = .78). At the second wave, students in group 1 (M = 3.06, SD = .82) scored significantly higher on analysing than their peers in group 2 (M = 2.89, SD = .75).

Tables 5 and 6 present the results from the sensitivity analyses. For the memorizing subscale, the models, assuming either MCAR or MAR, indicated a significant decrease over time, combined with variance in intercepts but not in slopes. However, the models assuming MNAR did not confirm a declining trend over time. The H&G model estimated that the general trend was insignificant, due to the fact that students who dropped out of their studies at a certain time were found to remain constant on this learning strategy subscale (*b* = .043, *se* = .054, *p* = .43; not in table). The estimates from the neighbouring and available case models also suggested an absence of change over time. In summary, the models, relying on different assumptions regarding missingness, disagreed on whether or not there was average growth.

**Table 5:** Parameter estimates and standard errors for the growth models for the scales ‘memorizing’ and ‘lack of regulation’.

	Mean intercept	Mean slope	Intercept variance	Slope variance	Covariance
**Memorizing°**					
MCAR					
Listwise deletion (LD)	3.318 (.054)***	-.083 (.026)**	.409 (.087)***	.032 (.033)	
MAR					
Maximum Likelihood (ML)	3.278 (.027)***	-.056 (.020)**	.415 (.071)***	.016 (.032)	
Multiple Imputation (MI)	3.283 (.028)***	-.059 (.024)*	.414 (.073)***	.015 (.032)	
ML with auxiliary variables (MLaux)	3.277 (.027)***	-.063 (.031)*	.398 (.069)***	.009 (.032)	
MI with auxiliary variables (MIaux)	3.286 (.030)***	-.066 (.032)*	.368 (.125)***	.005 (.031)	
MNAR					
Hedeker & Gibbons (H&G)	3.274 (.027)***	-.002 (.035)	.454 (.040)***	.029 (.018)	
Neighboring Case	3.274 (.027)***	-.003 (.037)	.454 (.040)***	.029 (.019)	
Available Case	3.274 (.027)***	-.040 (.024)	.454 (.040)***	.029 (.019)	
**Lack of regulation°**					
MCAR					
Listwise deletion (LD)	2.569 (.053)***	-.134 (.025)***	.350 (.077)***	.015 (.030)	
MAR					
Maximum Likelihood (ML)	2.869 (.026)***	-.181 (.020)***	.440 (.062)***	.051 (.026)	-.029 (.036)
Multiple Imputation (MI)	2.876 (.026)***	-.188 (.022)***	.446 (.059)***	.054 (.025)*	-.024 (.036)
ML with auxiliary variables (MLaux)	2.868 (.026)***	-.103 (.031)**	.464 (.061)***	.062 (.027)*	-.025 (.036)
MI with auxiliary variables (MIaux)	2.890 (.028)***	-.150 (.030)***	.522 (.064)***	.080 (.025)**	-.061 (.036)
MNAR					
Hedeker & Gibbons (H&G)	2.866 (.026)***	-.141 (.034)***	.403 (.036)***	.030 (.017)	
Neighboring Case	2.865 (.026)***	-.112 (.037)**	.401 (.035)***	.030 (.016)	
Available Case	2.865 (.026)***	-.124 (.024)***	.401 (.035)***	.030 (.016)	

*** *p* < .001

** *p* < .01

* *p* < .05

For the LD model: N = 225, for all other models: N = 1072

*Note*: The auxiliary variables were gender; prior education (general, technical or vocational); study track in higher education; whether or not students had started the first year in that university college anew; whether or not they had followed a non-delayed study trajectory; and the grade point average for each year.

**Table 6:** Parameter estimates and standard errors for the growth models for the scale ‘analysing’.

	Mean intercept	Mean slope	Intercept variance	Slope variance	Covariance
MCAR					
Listwise deletion (LD)	3.059 (.053)***	.009 (.026)	.340 (.079)***	.033 (.031)	
MAR					
Maximum Likelihood (ML)	2.890 (.024)***	.049 (.020)*	.350 (.058)***	.033 (.026)	
Multiple Imputation (MI)	2.901 (.027)***	.039 (.021)	.341 (.059)***	.030 (.027)	
ML with auxiliary variables (MLaux)	2.890 (.025)***	-.018 (.030)	.358 (.058)***	.035 (.027)	
MI with auxiliary variables (MIaux)	2.902 (.029)***	-.032 (.034)	.346 (.056)***	.033 (.027)	
MNAR					
Hedeker & Gibbons (H&G)	2.890 (.025)***	.039 (.034)	.360 (.033)***	.032 (.016)*	-.006 (.019)
Neighboring Case	2.890 (.025)***	.027 (.036)	.359 (.035)***	.032 (.016)*	-.006 (.019)
Available Case	2.890 (.025)***	.021 (.023)	.359 (.035)***	.032 (.016)*	-.006 (.019)

*** *p* < .001

** *p* < .01

* *p* < .05

For the LD model: N = 222, for all other models: N = 1071

*Note*: The auxiliary variables were gender; prior education (general, technical or vocational); study track in higher education; whether or not students had started the first year in that university college anew; whether or not they had followed a non-delayed study trajectory; and the grade point average for each year.

Concerning the lack of regulation subscale, the mean intercept was estimated to be significantly higher for the MAR and MNAR models compared to the LD model (95% CI LD 2.46–2.67; CI ML 2.823–2.92, obtained by using the CINTERVAL statement under OUTPUT, see S1 File. Please note that the CINTERVAL command cannot be used for the multiple imputation models.). The estimate of the mean slope did not significantly differ between the models; neither did the intercept variance. Next to this, whilst the LD model did not detect slope variance, the estimates for the ML model were at the verge of significance (*var slope* = .051, *se* = .026, *p* = .056) and those of the MI, MLaux and MIaux models reached significance (see Table 5). However, the MNAR models did not confirm this differential growth. Consequently, the LD model differed from the MAR models on the mean intercept and on the slope variance, whilst the MNAR and MAR models disagreed regarding the last parameter.

For the third subscale, analysing, we noted three differences. Firstly, the MAR and MNAR models estimated the mean intercept to be significantly lower compared to the LD model (95% CI LD: 2.96–3.16; CI ML: 2.84–2.94). Secondly, the LD, MI, MLaux and MIaux techniques and MNAR models estimated the mean slope to be not significantly different from zero. The ML estimates, on the other hand, suggested a positive mean slope over time. Thirdly, the MNAR models detected a significant variance in slopes, while the LD and MAR models did not.

## Discussion

Invariably, longitudinal studies have missing data. In practice, participants with missing data are often discarded, mostly by applying LD, which assumes an MCAR mechanism (see Table 1). On the other hand, methodologists recommend that a sensitivity analysis be conducted by estimating models that assume that missingness is related to either the study’s variables (MAR), or to the value, which would have been observed had the student provided data (MNAR). To help bridge the gap between methodological recommendations and applied research in the educational and psychological domain, the study provides a tutorial example of sensitivity analysis on a non-simulated data set. For this, the growth in three learning strategies (memorizing, analysing and lack of regulation) during higher education was estimated by using eight missing data techniques, which assumed respectively MCAR, MAR and MNAR (see Table 1). In the following paragraphs, the guidelines for reporting results from sensitivity analysis will be provided and applied to the tutorial example.

For the memorizing subscale, the MCAR assumption was not rejected. Nonetheless, as summarized in Table 7, the models suggested substantively different results. The LD model and the MAR models indicated a significant decline in memorizing over time, while the models assuming MNAR did not confirm this. These differences underline that the need for estimating models assuming MAR and MNAR and for conducting a sensitivity analysis is irrespective of finding significant results on the Little’s MCAR test or not [30].

**Table 7:** Summary of results from sensitivity analysis.

	Mean intercept	Mean slope	Intercept variance	Slope variance
Memorizing	=	Significant: LD, MAR; Not significant: MNAR models.	=	=
Lack of regulation	LD<MAR & MNAR	=	=	Significant: MI, MLaux & MIaux (ML at the verge);Not significant: LD & MNAR.
Analysing	LD>MAR & MNAR	Significant: ML;Not significant: other models.	=	Significant: MNAR;Not significant: LD & MAR.

*Note*: “ = “ signifies that there were no differences between the results of the different models; **“**LD<MLaux” means that the estimate is larger for the MLaux model than the LD model; “Significant: MI” indicates that the estimate is significant for the MI model; “Not significant: LD” means that in the LD model, the estimate did not result significant

This detected difference between the models brings the question to the fore on how to report these contradictory findings. Table 8 summarizes the guidelines presented in the methodology literature [2, 12, 13, 22, 24, 30]. For the memorizing scale, results in terms of the presence or absence of a significant decline in this learning strategy did not confirm one another (Table 8, option 1). Consequently, it is recommended that the researcher presents all models assuming MAR and MNAR. Next, taking prior knowledge, contextual information and possibly further information on the dropout mechanism into account [2, 13], the researcher has to choose the model based upon the assumptions with which he/she is most comfortable [22, 24, 30].

**Table 8:** Guidelines for reporting the results from sensitivity analysis.

	Result	How to report?
1	Models assuming MAR ≠ Models assuming MNAR	Present MAR and MNAR; Cautiously choose; Present findings cautiously.
2	Models assuming MAR ≈ Models assuming MNAR	MAR models in detail; Add: Not contradicted by MNAR.
3	ML ≠ MI, MLaux, MIaux & MNAR	Present MAR & MNAR models; Opt for the MI, MLaux, MIaux & MNAR results.

This choice is not an easy one, certainly in cases where further information on the dropout mechanism is lacking, or when it is unclear. Recall that the data do not provide guidance as to whether or not the MAR and/or MNAR mechanisms hold [11, 12]. Moreover, MNAR models may produce wrong results when their assumptions are violated [12, 15, 21]. However, the same goes for models assuming MAR: when MNAR data are modelled using MAR techniques, the results may also be biased [2, 8, 15]. As stated by Molenberghs et al. [2], rather than informing us on the adequacy of the MNAR model, the “…MNAR analysis may tell us about inadequacies of the original model” (p. 541), in the form of the MAR model. In other words, when sensitivity analysis reveals contradictory results, this decreases the confidence in the results of the MAR model. Hence the need to present both models assuming MAR and MNAR, and clarifying which assumptions appear most plausible, and to interpret the results cautiously.

Applying these guidelines to the results for the mean slope of memorizing using MAR and MNAR models, both models should be presented. Taking into account that learning strategies predict dropout [53] and that there are large time intervals between two waves in this study, making it possible that a change in learning strategies was unobserved, we assess the MNAR mechanism to be plausible here. Thus, we opt to refrain from stating that students decrease their degree of memorizing. Rather, we conclude that students continuing in higher education do reduce their reliance on memorizing strategies, whilst for those dropping out after the second wave, the trend is unclear and requires further research.

Concerning the subscale lack of regulation, LD underestimated the mean intercept, which was in line with the rejection of the MCAR assumption for this subscale at the first wave. This finding can be related to prior research that detected that students scoring higher on lack of regulation were more likely to drop out of higher education [53].

Regarding the mean slope, the results of the models assuming MAR are in line with those assuming MNAR (see Table 8, option 2): all describe a decline in lack of regulation over time. It is then suggested that researchers should report the results from models assuming MAR and that they are advised to add that models assuming MNAR, do not contradict these findings. The fact that this result holds up in a sensitivity analysis, increases the confidence with which we can interpret the finding: they can be considered more robust [2, 12].

Though the estimates for the mean slope did not vary between models for the lack of regulation scale, the estimates of the slope variance did (see Table 7). The LD and models assuming MNAR did not detect significant slope variance, whilst the models assuming MAR did. Given that the MAR and MNAR models do not speak with one voice (see Table 7, option 1), both models assuming MAR and MNAR should be presented. For the reasons stated above (effect of learning strategies on dropout chances and the large time intervals), we consider the MNAR assumption to be plausible for this given dataset. As such, we would refrain from concluding that there was slope variance.

For the third learning strategies scale, the analyzing subscale, three differences were noted. First, given that the MCAR assumption was disproven at the first wave, LD overestimated the mean intercept. Second, the ML results suggested a significant mean slope whilst those from MI, MIaux and MLaux did not (see Table 8, option 3). Although Jeličić et al. [65] reported on a comparable finding, the difference between the ML and MI estimates is disconcerting. If the set of cases and the used variables are the same, and if the number of imputed datasets is sufficiently large (here, *m* = 100), the ML and MI models should produce equivalent parameter estimates [5, 35, 44]. Yet, increasing the number of imputed datasets to 2,000 did not annul the difference between the ML and MI results.

One possible explanation was a violation of the multivariate normal distribution to which ML was found to be more sensitive than MI [65, 66]. However, there is a lack of guidelines as to whether, in this case, ML or MI is to be trusted more [65]. Therefore, a suggestion for practice could be to estimate all four MAR models when auxiliary variables are available, and both the ML and MI models when they are unavailable. Reporting on these findings (see Table 8, option 3), results from models assuming MAR or MNAR are to be presented. Those from the MI, MLaux, MIaux and MNAR models seem more plausible here, given that they confirmed one another.

A third difference in the results of the analysing scale concerns the slope variance. The MNAR models indicated differential growth, whilst the MAR models did not (see Table 8, option 1). Again, the estimates of both models assuming MAR and MNAR should be reported. Though the MNAR assumption seems plausible for this dataset, we take into account that the estimates for the slope variance only just achieved significance. Thus, to avoid type I error (i.e., stating there is differential growth over time when, in reality, there is not), we opt for the results of the models assuming MAR (no slope variance). Moreover, we suggest further research is necessary.

Three more general implications for the analysis of longitudinal data arise from these results. Firstly, the various models assuming different missing data mechanisms assuming MAR and MNAR led to substantively different conclusions. This implies that sensitivity analysis proved valuable in the present case. Regarding this, we would like to point out that researchers differ in their views regarding under which circumstances research practitioners in educational and psychological sciences should consider sensitivity analysis. Given that the MAR and MNAR assumptions cannot be tested [11, 12] in everyday research practice, one cannot rule out MNAR missingness for a given dataset. Some methodologists indicate that this absence of information is in itself enough to underpin the need for sensitivity analysis [2, 12, 13, 15]. In other words, when confronted with missing data, sensitivity analysis should be conducted.

Others have argued that the chances of data violating the MAR assumptions to such a degree as to impact the results obtained are slim [35, 38]. Moreover, for growth models, the bias is more pronounced for variance or covariances than for the mean slope [8], though the latter is often more relevant for the research practitioner. Adding this to the fact that MNAR models have a set of untestable assumptions themselves [12, 15, 31], one may be inclined to conduct sensitivity analysis only when there is a strong suspicion about data being MNAR [38]. Until the methodology literature can offer the applied researcher definitive guidance, we suggest conducting sensitivity analysis whenever MNAR appears plausible based on contextual information or prior knowledge, such as in the present case, the link between study success and dropout in higher education and students’ learning strategies [51, 52, 53].

Secondly, the LD approach often generated different estimates than did the models assuming MAR or MNAR. When the MCAR assumption was disproved for the first wave, the mean intercept was either over- or underestimated. As shown repeatedly in simulation studies [18, 27, 39], the results from the LD missing data technique were “…inadequate at best, misleading at worst” [65] (p. 819). LD should therefore be refrained from, in favour of models assuming MAR.

Thirdly, the estimates from MLaux and MIaux differed little from the models without auxiliary data. This concurs with Graham’s [32] point of view that, when all measures of the variable are included in a latent growth analysis, “…then the incremental benefit of other potential auxiliary variables is likely to be small” (p. 570). To further underpin the suggestions for research practice, simulation studies on whether or not to include auxiliary variables in latent growth analysis are welcome. Such studies would preferably also examine the impact of specific types of auxiliary variables that may increase bias [63] and balance this threat with their possible advantage of turning an MNAR missingness situation into MAR [10, 31, 44]. Moreover, the simulation studies on whether or not to include auxiliary variables that are little or unrelated to the variable under consideration relied on regression analysis [35, 64]. As such, it is unclear for practice whether the suggestions provided in these studies also hold for growth models. In our particular case, including or omitting variables that were little or not related to the variables under consideration did not matter. For the memorizing, analyzing and lack of regulation scale there were respectively 4, 6 and 7 variables that correlated at least .10 with the learning strategy at each of the 3 waves. When the MLaux and MIaux models were re-estimated with only these auxiliary variables, the results were very similar to those provided in Table 1. These findings can however not be generalized and we concur with Thoemmes and Rose [63] “…that there is still a lot to be learned about the selection of auxiliary variables in missing data” (p. 450). We believe this is especially true for more complex models such as growth models.

It has to be acknowledged that this study exhibited a number of limitations. Firstly, the results from this study cannot be generalized to other longitudinal studies or, specifically, to studies on the change in learning strategies during higher education. The issue of missing data is characteristic of each study and of each dataset. Consequently, each study warrants sensitivity analysis to assess whether or not the missing data technique influence the findings on longitudinal change.

Secondly, the longitudinal data set used contained only three waves of data. As a consequence, the differences between the H&G model and the other two PM models are very limited. This is not surprising given that in the H&G model subgroups 2 and 3 were grouped, while in both the neighbouring and available case models, the growth estimates from subgroup 2 were used to estimate the growth trend for subgroup 3. With datasets containing more waves, the drawback of the H&G model in treating all respondents with incomplete data as being alike might be more pronounced.

Thirdly, administrative data provided us with information on the registration of students in each of three academic years. This allowed us to discern *true* dropout patterns. When this data is not available, researchers need to construct plausible dropout patterns based on the observed data. However, as missing data is a mixture of nonresponse and attrition, it can be difficult to discern if and when a student has dropped out. In such cases, latent class PM models can be more adequate given that the pattern of missing data is not constrained equally to the dropout time (as with regular PM models) but is related to it in terms of probability [22, 23, 67].

Notwithstanding this study’s constraints, we hope to have provided a clear tutorial case with regard to applying sensitivity analysis in the case of latent growth analysis. It is apparent from the results that the choice of missing data technique influences the substantive conclusions arrived at. This underscores the need to conduct sensitivity analysis when missing data may be related to the concept under consideration and to report the findings according to the guidelines provided.

## Supporting information

S1 File Appendix. (DOCX)

S2 File Memorizing. (CSV)

S3 File Analysing. (CSV)

S4 File Lack Of Regulation. (CSV)
