# Supplementary material for: To what degree does the missing-data technique influence the estimated growth in learning strategies over time? A tutorial example of sensitivity analysis for longitudinal data
Source: PLoS One. 2017 Sep 13;12(9):e0182615. doi: 10.1371/journal.pone.0182615 (PMC5597092; doi:10.1371/journal.pone.0182615)
Supplement: S1 File — (DOCX) [file pone.0182615.s001.docx]

## Appendix: Example M*plus* Syntaxes for the Memorizing Scale

For each model, the syntax is provided along with explanation. This explanation is preceded by the “!” symbol, since Mplus does not read lines which start with “!”.

###

### **MCAR Assumption**

#### **Growth model on the listwise deleted sample.**

DATA: file =

C:\Users\Desktop\Missingness\Memorizing\Memorizing.csv;

! the following indicates that you will only use those respondents for whom complete data are !available

LISTWISE = ON;

VARIABLE:

! description of the variables in the dataset, in this case the students' id number, their score at

!the three waves, 15 auxiliary variables and patterns of missingness

VARIABLE:

names are id y1 y2 y3 aux1 aux2 aux3 aux4 aux5 aux6 aux7 aux8

aux9 aux10 aux11 aux12 aux13 aux14 aux15 dropout pattern;

! indicate which variables you will use in the analysis

usevariables = y1 y2 y3;

! indicate how Mplus can detect whether data are missing, you can define the value yourself

missing = all (999);

MODEL:

! the actual analysis model, here a growth model with unequal time intervals

!(here, 14 months between wave 1 and 2 and 12 months between wave 2 and 3)

! icept refers to the mean intercept, linear refers to the mean slope

icept linear | y1@0 y2@1.17 y3@2.17;

! ask for the TECH4 output which you can consult if there are problems with the model

! the CINTERVAL gives you confidence intervals for the estimates

OUTPUT: TECH4; CINTERVAL;

! Note: you do not need to define that the analysis needs to use maximum likelihood as it is the !default in Mplus for this analysis

###

### **MAR Assumption**

#### **Maximum likelihood.** From this model onwards, all respondents are used to estimate the model, so also those with missing data

DATA: file =

C:\Users\Desktop\Missingness\Memorizing\Memorizing.csv;

VARIABLE:

names are id y1 y2 y3 aux1 aux2 aux3 aux4 aux5

aux6 aux7 aux8 aux9 aux10 aux11 aux12

aux13 aux14 aux15 dropout pattern;

usevariables = y1 y2 y3;

missing = all (999);

MODEL:

icept linear | y1@0 y2@1.17 y3@2.17;

OUTPUT: TECH4; CINTERVAL;

#### **Multiple imputation.**

***Step 1: Imputation***

DATA: file = C:\Users\Desktop\Missingness\Memorizing\Memorizing.csv;

VARIABLE:

names are id y1 y2 y3 aux1 aux2 aux3 aux4 aux5

aux6 aux7 aux8 aux9 aux10 aux11 aux12

aux13 aux14 aux15 dropout pattern;

usevariables = y1 y2 y3;

missing = all (999);

! in the Data imputation command you state that variables y1-y3 need to be imputed and that you !require 100 imputed datasets

DATA IMPUTATION:

impute = y1-y3 ;

ndatasets = 100;

save = memoimp*.dat;

ANALYSIS: TYPE = BASIC;

OUTPUT: TECH8;

***Step 2: Analysis and pooling.*** The memoimplist.dat file, made in step 1, is now used for the analysis

DATA: FILE = C:\Users\Desktop\Missingness\Memorizing\memoimplist.dat;

TYPE = IMPUTATION;

VARIABLE:

! the names statement below refers to those variables in the 100 created datasets. Given that in !step1 y1-y3 were imputed, there are now three variables

names are y1 y2 y3;

usevariables = y1 y2 y3;

ANALYSIS: ESTIMATOR = ML;

MODEL: i s | y1@0 y2@1.17 y3@2.17;

OUTPUT: TECH4;

#### **Maximum likelihood with auxiliary variables.**

DATA: file = C:\Users\Desktop\Missingness\Memorizing\Memorizing.csv;

VARIABLE:

names are id y1 y2 y3 aux1 aux2 aux3 aux4 aux5

aux6 aux7 aux8 aux9 aux10 aux11 aux12

aux13 aux14 aux15 dropout pattern;

usevariables = y1 y2 y3;

! the auxiliary variables will be used while estimating the model. This can be done using

! auxiliary = (m) …

auxiliary = (m) aux1 aux2 aux3 aux4 aux5 aux6 aux7 aux8 aux9 aux10 aux11 aux12

aux13 aux14 aux15;

missing = all (999);

MODEL:

icept linear | y1@0 y2@1.17 y3@2.17;

OUTPUT: TECH4; CINTERVAL;

#### **Multiple imputation with auxiliary variables**

***Step 1: Imputation.*** For this model, you need the auxiliary variables in your csv-file.

DATA: file =

C:\Users\Desktop\Missingness\Memorizing\Memorizing.csv;

VARIABLE:

names are id y1 y2 y3 aux1 aux2 aux3 aux4 aux5

aux6 aux7 aux8 aux9 aux10 aux11 aux12

aux13 aux14 aux15 dropout pattern;

! the variables which you wish to use as auxiliary variables should be in the usevariables !statement here. There is also an auxiliary statement possible, but this is for variables you want to !include the imputed data sets, but are not used to create the imputed data sets:

usevariables = y1 y2 y3 aux1 aux2 aux3 aux4 aux5 aux6 aux7 aux8 aux9 aux10 aux11

aux12 aux13 aux14 aux15;

missing = all (999);

DATA IMPUTATION:

impute = y1-y3 ;

ndatasets = 100;

! by using memoimpB*.dat instead of memoimp*.dat, you do not overwrite the datasets created !for the multiple imputation model without auxiliary variables

save = memoimpB*.dat;

ANALYSIS: TYPE = BASIC;

OUTPUT: TECH8;

***Step 2: Analysis and pooling.***

! make sure to refer to memoimpBlist.dat as to avoid accidentally using the datasets created for !the multiple imputation model without auxiliary variables

DATA: FILE = C:\Users\Desktop\Missingness\Memorizing\memoimpBlist.dat;

TYPE = IMPUTATION;

VARIABLE:

names are y1 y2 y3 aux1 aux2 aux3 aux4 aux5 aux6 aux7 aux8 aux9

aux10 aux11 aux12 aux13 aux14 aux15;

usevariables are y1 y2 y3;

ANALYSIS: ESTIMATOR = ML;

MODEL: i s | y1@0 y2@1.17 y3@2.17;

OUTPUT: TECH4;

### **MNAR Assumption**

#### **Hedeker & Gibbons.** For this model, you need a dummy in your csv file discerning 2 groups. Here, the dropout variable indicates whether a student progressed normally throughout their 3 years of study (0, completers) or not (1, dropout)

DATA: file =C:\Users\Desktop\Missingness\Memorizing\Memorizing.csv;

VARIABLE:

VARIABLE:

names are id y1 y2 y3 aux1 aux2 aux3 aux4 aux5

aux6 aux7 aux8 aux9 aux10 aux11 aux12

aux13 aux14 aux15 dropout pattern;

! though there is only one dropout variable in the dataset, you refer to both dropout and dropout2 !in the usevariables line. The dropout2 variable will be created later.

usevariables = y1 y2 y3 dropout dropout2;

missing = all (999);

nominal are dropout2;

! creating the dropout2 variable

define: dropout2 = dropout;

ANALYSIS:

estimator = ml;

MODEL:

icept slope | y1@0 y2@1.17 y3@2.17;

y1-y3 (1);

[icept] (b00);

[slope] (b10);

icept on dropout (b02);

slope on dropout (b12);

[dropout2#1] (logit);

MODEL CONSTRAINT:

! first, you define new parameters

new(pic pid iceptc slopec iceptd sloped iceptavg slopeavg );

! computing pattern proportions

pic = exp(logit)/(exp(0) + exp(logit));

pid = exp(0)/(exp(0) + exp(logit));

! the estimates for the completers

iceptc = b00;

slopec = b10;

! the estimates for the dropouts

iceptd = b00 + b02;

sloped = b10 + b12;

! calculating the average estimates across patterns, it are these average estimates that you report

iceptavg = pic*iceptc + pid*iceptd;

slopeavg = pic*slopec + pid*sloped;

OUTPUT: sampstat; CINTERVAL;

#### **Model with neighboring case restriction.**

#### For this model, you need a variable in your csv-file discerning 3 groups. Here, the pattern variable indicates whether a student was in a non-delayed trajectory (pattern 1), registered up to the second year (pattern 2, dropout after wave 2) or registered only in the first year (pattern 3, dropout after wave 1)

DATA: file =C:\Users\Desktop\Missingness\Memorizing\Memorizing.csv;

VARIABLE:

names are id y1 y2 y3 aux1 aux2 aux3 aux4 aux5

aux6 aux7 aux8 aux9 aux10 aux11 aux12

aux13 aux14 aux15 dropout pattern;

usevariables are y1 y2 y3 ;

missing are all (999);

! missing data patterns are defined as three known classes

classes = patt(3);

knownclass = patt(pattern = 1 pattern = 2 pattern = 3);

ANALYSIS:

! given you have different classes, you need mixture modeling

type = mixture;

MODEL:

%overall%

icept slope | y1@0 y2@1.17 y3@2.17;

y1-y3 (1);

[patt#1] (p1logit);

[patt#2] (p2logit);

! models per pattern

! pattern 1

%patt#1%

[icept] (p1i);

[slope] (p1s);

! pattern 2

%patt#2%

[icept] (p2i);

[slope] (p2s);

! pattern 3

%patt#3%

[icept] (p3i);

[slope] (p3s);

MODEL CONSTRAINT:

! restraining the mean slope for the group with only 1 data point (pattern 3) to the mean slope of t

!who are registered up to the second year (pattern 2), which are viewed here as ‘neighbour’

p3s = p2s;

! calculating the iceptavg and slopeavg, which are reported

new(c1prop c2prop c3prop iceptavg slopeavg );

c1prop = exp(p1logit)/(exp(0) + exp(p1logit) + exp(p2logit));

c2prop = exp(p2logit)/(exp(0) + exp(p1logit) + exp(p2logit));

c3prop = exp(0)/(exp(0) + exp(p1logit) + exp(p2logit));

iceptavg = c1prop*p1i + c2prop*p2i + c3prop*p3i;

slopeavg = c1prop*p1s + c2prop*p2s + c3prop*p3s;

OUTPUT: sampstat; CINTERVAL;

#### **Model with available case restriction.** Compared to model with neighboring case restriction, only the restriction in the model constraint section differs.

MODEL CONSTRAINT:

! restraining the mean slope for the group with only 1 data point (pattern 3) to the weighted !average of the mean slopes for pattern 1 and 2. To do so, you need the number of students !showing pattern 1 and 2 (here, respectively 395 and 184)

p3s = (395/(395+184))*p1s + (184/(395+184))*p2s ;
